# Biosorption of Uranium and Rare Earth Elements Using Biomass of Algae

**DOI:** 10.1155/2008/706240

**Published:** 2008-12-01

**Authors:** Nobuo Sakamoto, Naoki Kano, Hiroshi Imaizumi

**Affiliations:** ^1^Graduate School of Science and Technology, Niigata University, Ikarashi 2-nocho 8050, Nishi-Ku, Niigata 950-2181, Japan; ^2^Faculty of Engineering, Niigata University, Ikarashi 2-nocho 8050, Nishi-Ku, Niigata 950-2181, Japan

## Abstract

In order to investigate the behavior of rare earth elements (REEs) and uranium (U) in marine organism, the concentrations of REEs and U in some brown algae samples taken on the coast of Niigata Prefecture were determined. In addition, laboratory model experiment to uptake these elements using living and dried algae (*Undaria pinnatifida* and *Sargassum hemiphyllum*) was also carried out to survey the uptake and bioaccumulation mechanism of REEs and U in algae. Consequently, the following matters have been mainly clarified. (1) The order of the concentration of REEs for each organ in *Sargassum hemiphyllum* is “main branch” > “leaf” > “vesicle,” however for U, the order is “leaf” > “vesicle” > “main branch.” (2) The concentration of REEs in *Sargassum hemiphyllum* may be strongly affected by suspended solid in seawater. (3) The uptake and/or accumulate mechanism of REEs in brown algae may be different from that of U.

## 1. INTRODUCTION

Thorium (Th) and uranium (U) are natural radionuclides and are widely distributed in nature due to the nuclear power production as well as to a number of human activities (e.g., mining, production, and use of phosphate fertilizers, copper metallurgy, and military activities) [1, 2]. These elements are possible harmful pollutants in the environment. Hence, investigating the concentrations of Th and U in the environment is significant from a radiation and pollution protection viewpoints [3].

On the other hand, determination of rare earth elements (REEs) in marine organism such as seaweed is important in studying the extent of these REEs to marine environment. REEs are considered important tracers for studying the circulation of materials in the biosphere [4, 5]. Our knowledge about the environmental behavior of the REE has increased because of the development of new analytical techniques.

Marine organism such as seaweed is well known to concentrate metals and has been used as a monitor of seawater pollution [6–9]. Moreover, biosorption studies using living biomass including seaweed have been widely performed in large parts of the world recently [10–13].

It is known that alginate is an exopolymer extracted mainly from brown algae and various bacteria that have been used both as an immobilization material and as a biosorbent of several heavy metals. Moreover, alginate is one of the constituents of the cell walls of brown algae, and it consists of mannuronic and guluronic acid monomers. Oligopolymeric blocks of guluronic acid show a high selectivity for heavy metal ions [14, 15].

The coasts in the vicinity of Niigata Prefecture (including Sado Island) are located in the geographic position where they are affected by both the cold ocean and the warm ocean current systems, so many kinds of marine organisms occur. However, in studies of seaweed in Japan, most of previous works have been conducted on the Pacific coast, and the chemical data for seaweed on the coast of the Japan Sea are extremely limited (particularly regarding the determination of REEs, Th, and U).

Therefore, we have determined the concentrations of REEs [16], Th, and U [17] in various kinds of seaweed taken on the coast of Niigata Prefecture, and consequently the following matters have been mainly clarified. The concentrations of REEs in seaweed species from Niigata Prefecture were generally about 10^2^-10^3^ times higher than those in seawater, and the enrichment factors of REEs were larger in heavy REE (HREE) than light REE (LREE). A significant concentration difference of REEs, Th, and U was found among species even in the same phylum. The concentration of U was generally higher in brown algae and was the highest in *Undaria pinnatifida*.

Considering the above mentioned, the concentrations of REEs and U in some brown algae samples taken on the coast of Niigata Prefecture were determined in present work to investigate the behavior and/or the biological concentration of REEs and U in marine organism in more detail. Furthermore, to survey the uptake and bioaccumulation mechanism of REEs and U in algae, laboratory model experiment to uptake REEs and U using living and dried algae was also done. Among REEs, lanthanides (i.e., La-Lu) were selected in analysis of environmental samples (seaweed and seawater). The lanthanides (La-Lu), particularly, are of great interest because of their similar chemical behavior that allows them to be used as a tracer of a wide variety of geochemical processes. The REE pattern, where the abundance of each lanthanide relative to that of a chondrite or shale is plotted on a logarithmic scale against the atomic number, is therefore regarded as a “finger-print” of a geological sample [18, 19]. In case of laboratory model experiment, La, Eu, and Yb were selected as the representatives of light REE (LREE), medium REE (MREE), and heavy REE (HREE) based on Diniz and Volesky's work [11]. Two species of brown algae: *Sargassum hemiphyllum* and *Undaria pinnatifida*, were chosen in this model experiment, because they are general species in Japan (particularly, *U. p.* is used as a part of daily diet in Japan), and easily found at almost all sampling locations on the coast in Niigata Prefecture.

## 2. EXPERIMENTAL

### 2.1. Reagents

Lanthanide elements and U standard solutions used for making the calibration curve were prepared by diluting the standard solutions (XSTC-1 for REEs and XSTC-13 for 31 elements including U; both 10 mg dm^−3^ 5% HNO_3_ solutions) purchased from SPEX CertiPrep, Inc. (NJ, USA). Alginate acid was purchased from Acros Organics (NJ, USA). All other chemical reagents were purchased from Kanto Chemical Co., Inc. (Tokyo, Japan). All reagents used were of analytical grade, and deionized and distilled water was used.

### 2.2. Samples

**Figure 1:**
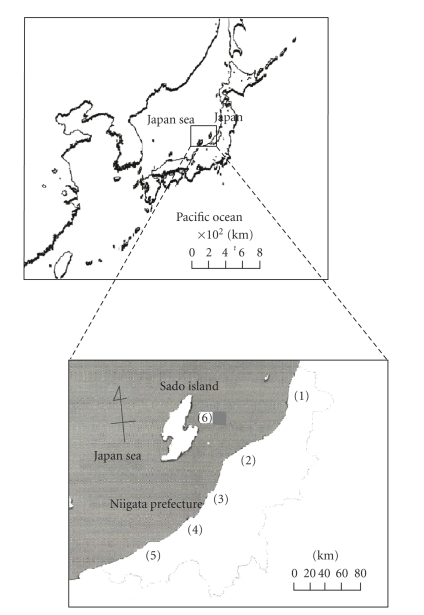
Location of sampling points. (1) Sampoku town (2) Niigata city(3) Izumozaki town (4) Kashiwazaki city (5) Itoigawa city (6) Sado city.

Five species of brown algae: *Dictyota dichotoma, Ecklonia stolonifera, Sargassum hemiphyllum* (abbreviated as *S. h.* below), *Sargassum honeri,* and *Undaria pinnatifida* (abbreviated as *U. p.* below), were collected along several coasts in Niigata Prefecture in Japan (Figure 1) from 2004 to 2007. All samples were sampled at rock reefs about 100 m from the coast. For example, *S. h.* grew on rocks in the littoral zone. On the other hand, *U. p.* grew on rocks between the littoral zone and the infralittoral zone. Each seaweed sample was washed in the surrounding seawater to remove attachment at sampling place and was well washed by filtered seawater and deionized water in our laboratory as outlined in the work of Kato et al. [20].

Seawater samples were also collected from each sampling point. Each seawater sample was filtered through a 0.45 *μ*m membrane filter immediately after sampling. The basic physical parameters such as water temperature, pH, electric conductivity (EC), oxidation-reduction potential (ORP), and dissolved oxygen (DO) of the samples were measured by a handy pH/COND and pH/DO meter (HORIBA, D-24, and D-25) at each sampling point.

## 3. ANALYTICAL METHOD

### 3.1. Determination of REEs and U in seawater samples

The preconcentration of lanthanides and U was carried out according to Takaku et al. [21], and the procedure is briefly described as follows. These elements in seawater samples were separated from matrix and concentrated by a chelate disk (47 *φ*mm) (Empore Sumitomo 3M Co., Tokyo, Japan). In the separation process, the disk was placed in an ordinary disk holder. Each sample was run through the disk after adjusting the pH to 3 by using ammonium acetate (CH_3_COONH_4_) and nitric acid (HNO_3_). Then, lanthanides and U on the disk were eluted by 1.5 mol dm^−3^ HNO_3_ (20 cm^3^). Quantitative recovery of REEs and U from seawater was determined by “addition and recovery testing”: adding the subject element of known concentration to a sample, and measuring the concentrations of the element in both this additional sample and a nonadditional sample (comparing the analytical result of this additional sample with that of nonadditional sample). It was confirmed that quantitative recovery of lanthanides and U in seawater samples was obtained. The suspended solid on membrane filter was dissolved with 10 cm^3^ HNO_3_, 5 cm^3^ HF and, a proper amount of H_2_O_2_ in a PTFE beaker. The sampling solution was evaporated to dryness. After that, the residue was dissolved with 50 cm^3^ of 1 mol dm^−3^ HNO_3_ again. After the preconcentration procedure, the concentrations of lanthanides and U in samples were measured with an ICP-MS (HP4500; Yokogawa Analytical Systems, Tokyo, Japan). The operating condition of ICP-MS (including the measured isotope) is the same as that in our previous work [17].

### 3.2. Determination of REEs and U in seaweed samples

Each seaweed sample was dried at 110°C for 24 hours, and was ashed at 550°C for 48 hours. In addition, dry *S. h.* sample was separated into some different organs such as leaf, main branch, and vesicle. The ash (ca. 0.5 g) and each organ of dry *S. h.* (ca. 1.0 g) were dissolved with 10 cm^3^ HNO_3_, 5 cm^3^ HF, and a proper amount of H_2_O_2_ in a PTFE beaker. The sample solution was evaporated to dryness. After that, the residue was dissolved again with 50 cm^3^ of 1 mol dm^−3^ HNO_3_. The decomposition of seaweed was based on the procedure described by Fu et al. [22] and Sakao et al. [6]. After the above-mentioned procedure, the concentrations of lanthanides and U in these samples were measured with ICP-MS.

### 3.3. Determination of alginate content in seaweed samples

The extraction of alginate was based on the work of Tamiya and Watanabe [23], and the procedure is briefly described as follows. *S. h.* and *U. p.* samples were washed with tap water and then dried at 50°C for 48 hours. Each sample (ca. 10 g) was shaken with 1 dm^3^ of 0.2 N H_2_SO_4_ for 24 hours. The solution was filtered through membrane (Filter Paper Qualitative 2, Advantec), and the residue was shaken with 1 dm^3^ of 1% Na_2_CO_3_ for 24 hours. The solution was filtered again and was stirred with 1 dm^3^ of ethanol to precipitate alginic acid as sodium alginate. Suspension was washed with 0.2-0.3 dm^3^ of ethanol and diethyl ether and was dried at 30°C for 24 hours. The weight of dried sodium alginate was measured.

### 3.4. Model experiment using fresh and dry seaweeds

Samples used in this model experiment were fresh *S. h.* and *U. p.* In addition to alginic acid, dried *U. p.* was also used as reference. Dried sample was washed by deionized water and dried for 48 hours at 50°C. Alginic acid was shaken in filtered seawater for 24 hours. Seawater used in this study was filtered by Advantec TCW-3N-PPSE filter. The initial concentration of lanthanides and U in seawater was adjusted approximately to 6 ppb by adding lanthanide and U standard solution. Biomass samples (ca. 0.8 g dried weight) and alginic acid (ca. 0.4 g) were shaken in 200 cm^3^ prepared seawater on prescribed time (10, 20, 30, 40, 50, 60, 120, 360, 720, 1080, 1440, and 1800 minutes) at 15°C. Afterwards, matrix ions such as Na and K were removed, and the subject elements such as lanthanide and U were concentrated by using chelating cation-exchange resin (BioRad Chelex 100 Resin). Initial and final concentrations of lanthanides and U in solution were also measured with ICP-MS. The uptake amount (*A*) of lanthanides and U due to each sample was estimated by subtracting the final concentrations (*C_f_*) from initial concentrations (*C_i_*) in the liquid phase expressed as the following equation:

(1) $$A\;=\;(C_{i}-C_{f})V/M\;\;m\;\;[\text{mole}\;\;{\text{g}}^{-1}],$$

where *V* is the volume of the solution (0.2 dm^3^), *M* is the atomic weight of each element, and *m* is the dry weight of each sample.

## 4. RESULTS AND DISCUSSION

### 4.1. Behavior of REEs and U in marine environment

The concentrations of lanthanides and U in five kinds of brown algae taken on the coast of Niigata Prefecture are shown in Table 1. The relative standard deviation (RSD) of three replicated analyses of each sample was less than 10%. In Table 1, the concentrations of elements in seaweed are expressed on the basis of dry weight (in addition to ash weight). From this table, it is noted that *U. p.* showed highest concentration of U in five samples. In regard to lanthanide elements, the concentrations of elements of even atomic numbers (i.e., Ce, Nd, Sm, Gd, Dy, Er, and Yb) were generally larger than those of neighboring elements of odd atomic numbers (i.e., La, Pr, Eu, Tb, Ho, Tm, and Lu), obeying Oddo-Harkins' law.

**Figure 2:**
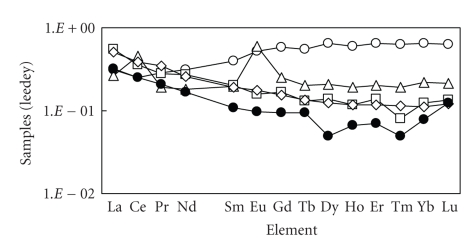
Normalized REE concentrations for brown algae samples (∘: Dictyota dichotoma, Δ: Ecklonia stolonifera, □ Sargassum hemiphyllum, ◊: Sargassum honeri, •: Undaria pinnatifida).

The concentrations of lanthanides in each sample were normalized to Leedey chondrite, and the relative concentrations are plotted in logarithmic scale against atomic numbers (i.e., REEs patterns). REEs patterns for five species of brown algae in this work were shown in Figure 2. In this figure, the average concentrations of each sample are indicated.

From this figure, the tendency of light REE (LREE) enrichment was generally found for many samples except *Dictyota dichotoma*. It is suggested that these samples are affected by crustal source (i.e., soil origins) to some extent. Moreover, large deviation of Eu in *Ecklonia stolonifera* is found. Most of the lanthanide elements have the valency of +3 on the earth, while only Ce and Eu are known to take different valencies; Eu may have valency of +2 besides +3 [24]. Then, the deviation of Eu in *Ecklonia stolonifera* may be closely related to the anomalous behavior of Eu (e.g., difference in solubility or stability of compounds) due to different valencies, although the cause of Eu deviation in this sample has yet to be sufficiently clarified in our work.

**Figure 3:**
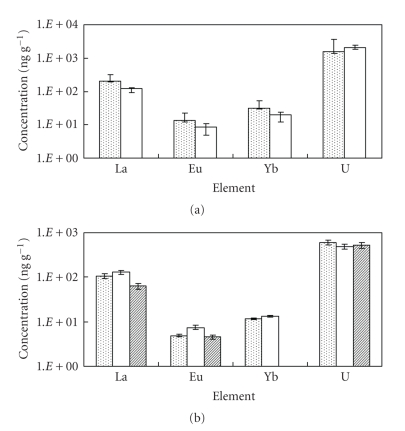
The concentrations of REEs and U in brown algae sample. (a) Bulk sample; (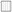): *S. h.*, (□): *U. p.*; (b) each organ in *S. h.*; (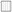): leaf, (□): main branch, (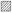): vesicle.

The concentrations of La, Eu, Yb, and U in *S. h.* and *U. p.* bulk samples were shown in Figure 3(a). Compared to *U. p.* sample, the concentrations of REEs (i.e., La, Eu, and Yb) in *S. h.* were comparatively higher, although the concentration of U is slightly smaller.

The concentrations of La, Eu, Yb, and U for each organ in *S. h.* are shown in Figure 3(b). From this figure, it is found that the order of the concentration of U is “leaf” > “vesicle” > “main branch.” On the other hand, the order of the concentration of REEs (i.e., La, Eu, and Yb) is “main branch” > “leaf” > “vesicle.” In case of REEs, the difference of accumulation among organs is remarkably observed. It is suggested that the degree of uptake and/or accumulation for each organ may strongly depend on the character of elements.

It is considered that alginate is significant for the uptake and/or accumulation of metals in brown algae. Hence, the content of alginate in *S. h.* and *U. p.* was determined (Table 2).

As shown in Table 2, no definite difference of alginate content between these two kinds of algae was observed, although the content of alginate in *U. p.* was slightly larger than that in *S. h.* Furthermore, Table 2 shows that the content of alginate comprises close to half of the representative brown algae.

**Figure 4:**
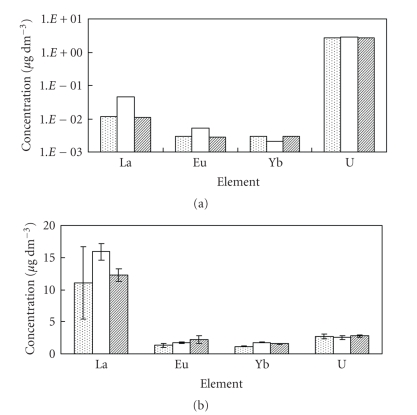
The concentrations of REEs and U in seawater samples. (a) Dissolved fraction; (b) suspended solid fraction. (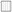): Itoigawa city, (□): Izumozaki town, (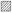): Kashiwazaki city.

The concentrations of La, Eu, Yb, and U in seawater samples are shown in Figure 4(a) (dissolved fraction) and Figure 4(b) (suspended solid fraction). From Figure 4(a), it is found that the concentration of U in seawater (dissolved fraction) is about 10^2^-10^3^ times higher than that of lanthanides. On the other hand, the concentration of La in suspended solid fraction in seawater is larger than that of Eu, Yb, and U. It is suggested that the concentration of REEs, particularly LREE (light REE) such as La in *S. h.* may be strongly affected by suspended solid in seawater.

In this work, the concentration factor is defined as the ratio of “the concentrations of elements in sample such as seaweed (ngg^−1^)” to “the concentrations of elements in seawater (*μ*g dm^−3^)” based on the work of Koyama et al. [7]. The average concentration factor of La, Eu, Yb, or U in *S. h.* and *U. p.* is shown in Table 3.

For both algae, the concentration factors of lanthanides (La, Eu, Yb) are much larger than those of U. In addition, it is noteworthy that the concentration factors of lanthanides are larger in *S. h.* than in *U. p.*, whereas those of U are smaller in *S. h.*

### 4.2. Behavior of REEs and U in model experiment

**Figure 5:**
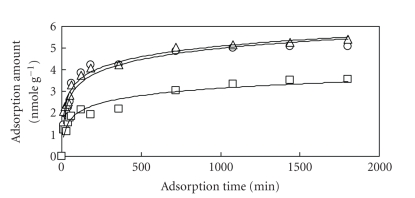
The amount of REEs adsorption to dried *S. h.* sample. (∘): La, (Δ): Eu, (□): Yb.

Time dependency of the amount of REEs (i.e., La, Eu, and Yb) adsorption to dry *U. p.* is shown in Figure 5. From this figure, it is found that 12 hours were required to achieve equilibrium. The amount of adsorption varies depending on each element, and the order of adsorption capacity was Eu ≥ La > Yb. This order was in accordance with that of Diniz's work [11]. They suggested that it may be attributable to the distribution coefficient and affinity to biomass. However, the adsorption behavior is similar among three elements. Based on this result, La was used as the representative of REEs in model experiment hereinafter.

**Figure 6:**
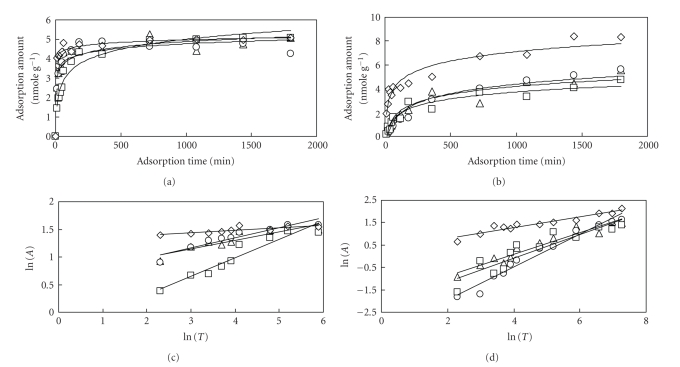
The amount of (a) La or (b) U adsorption to each sample. (∘): *S. h.*, (Δ): *U. p.*, (□): dry *U. p.*, (◊): alginic acid. The amount of La or U adsorption to each sample. (∘): *S. h.*, (Δ): *U. p.*, (□): dry *U. p.*, (◊): alginic acid ((c): La in logarithmical scale, (d): U in logarithmical scale).

The adsorption amount of La or U to each sample is shown in Figure 6(a) for La and Figure 6(b) for U. Figure 6(a) is replotted in logarithmic scale until the equilibrium is attained (Figure 6(c)). The adsorption amount of La on living biomass was similar to that on dry biomass. However, the difference of reaction rate between living biomass and dry biomass was remarkably observed. On the other hand, 24 hours were required to achieve equilibrium in case of the adsorption of U (Figure 6(b)). Figure 6(b) is also replotted in logarithmic scale until the equilibrium is attained (Figure 6(d)). From this figure, the difference of reaction rate of U among these samples was slightly found, however the difference was not as large as that of REEs.

The adsorption capacity of La and U on each sample is shown in Table 4. No large difference of adsorption capacity between *S. h.* and *U. p.* was observed for both elements, although *S. h.* showed slightly higher adsorption capacity than *U. p.* As shown in Figure 3(a), the concentration of La in *S. h.* is larger than that of *U. p.* However, large difference of adsorption capacity of La between two species was not observed in this model experiment.

In this model experiment, filtered seawater was employed. In other words, the effect of suspended solid (in which the concentration of La is large) is removed. That might be one reason for detecting no large difference of adsorption capacity of La between two species.

From Table 4, it can be also observed that the adsorption capacity of U was higher than that of La for both two algae. It is reported that alginate adsorbs divalent metal ion selectively [14, 25]. It is known that U exists in different forms depending on pH, and that at pH ≤ 4.3, U exists predominantly as monomeric species, UO_2_^2+^, and small amounts of UO_2_ (OH)^+^. At pH ≥ 5, colloidal or oligomeric species, that is, (UO_2_)_2_(OH)_2_^2+^, (UO_2_)_3_(OH)_5_^+^, (UO_2_)_4_(OH)_7_^+^, and (UO_2_)_3_(OH)_7_^−^, are formed [1, 26–28]. On the other hand, it is considered that REEs usually exist as trivalent ions in environmental waters and that organic complexes dominate at pH from 4 to 8, whereas carbonate complexes are the predominant species at alkaline pH ≥ 8 and REEs relatively tend to be precipitated at higher pH [29, 30].

Thus, higher adsorption capacity of U (than that of REEs) particularly found in alginate might be attributable to the chemical behavior of element in solution (e.g., pH-dependence of elements or stability of compounds), although further investigation of the uptake mechanism of elements is needed.

From this work, the behavior (or the uptake method) of REEs and U on seaweed could be clarified to some extent. The data obtained and the method used in this work can be useful for investigating the marine environment.

## 5. CONCLUSION

The concentrations of REEs and U in some brown algae samples taken on the coast of Niigata Prefecture were determined. In addition, chemical analysis per each organ is performed in *Sargassum hemiphyllum* (*S. h.*). Consequently, it is found that the concentrations of REEs (i.e., La, Eu, and Yb) in *S. h.* were comparatively higher than those in *Undaria pinnatifida* (*U. p.*), although the concentration of U in *S. h.* is slightly smaller. The order of lanthanides concentration in *S. h.* is “main branch” > “leaf” > “vesicle,” however for U, the order is “leaf” > “vesicle” > “main branch.” The concentration of REEs such as La in *S. h.* may be strongly affected by suspended solid in seawater.

Moreover, laboratory model experiment to uptake these elements using living and dried algae (*S. h.* and *U. p.*) was also carried out to survey the uptake and bioaccumulation mechanism of REEs and U in algae. Consequently, it is suggested that the uptake and/or accumulate mechanism of REEs in brown algae may be different from that of U due to the chemical behavior of element.

## Figures and Tables

**Table 1:** The concentrations (ng g^−1^) of lanthanide and U in brown algae taken on the coast of Niigata prefecture.

	Concentration/ng g^−1*^				
	*Dictyota dichotoma*(*n* = 2)	*Ecklonia stolonifera*(*n* = 1)	*Sargassum hemiphyllum*(*n* = 5)	*Sargassum honeri*(*n* = 5)	*Undaria pinnatifida*(*n* = 5)
La	(1.1–1.3) × 10^2^	1.0 × 10^2^	(1.9–2.5) × 10^2^	(1.4–3.0) × 10^2^	(0.93–1.3) × 10^2^
	35–38	20	38–53	33–66	30–48

Ce	(2.4–2.6) × 10^2^	4.4 × 10^2^	(3.3–4.0) × 10^2^	(3.1–4.4) × 10^2^	(1.9–2.8) × 10^2^
	74–79	92	71–1.0 × 10^2^	77–95	67–1.0 × 10^2^

Pr	38–40	26	(1.9–4.4) × 10^2^	37–60	(2.1–3.3) × 10^2^
	12-13	5.4	4.9–9.7	9.3–13	5.3–11

Nd	(2.1–2.4) × 10^2^	1.3 × 10^2^	(1.9–2.2) × 10^2^	(15–23) × 10^2^	92–1.5 × 10^−2^
	68–70	27	40–51	34–49	23–44

Sm	85–99	80	40–53	36–52	14–34
	28-29	16	10–13	8.2–12	3.6–12

Eu	41–49	3.1 × 10^2^	12–15	12–22	5.0–11
	13-14	64	2.7–3.9	2.6–4.8	1.9–3.9

Gd	(1.8-1.9) × 10^2^	1.5 × 10^2^	49–58	37–70	16–39
	55–62	31	10–15	8.0–15	3.8–12

Tb	30–36	1.1 × 10^2^	7.1–10	6.1–10	2.4–8.7
	9.7–10	23	1.4–2.4	1.3–2.2	6.0 × 10^−1^–3.1

Dy	(2.3–2.8) × 10^2^	1.4 × 10^2^	50–66	34–69	9.6–29
	77–80	28	11–16	9.4–15	3.4–9.9

Ho	53-54	1.2 × 10^2^	8.5–12	8.9–16	3.5–8.4
	16-17	24	1.9–3.2	2.0–3.5	9.0 × 10^−1^–2.8

Er	(1.5–1.8) × 10^2^	1.3 × 10^2^	31–40	13–22	13–24
	50–53	28	7.8–9.2	2.9–4.8	3.8–7.4

Tm	22–27	1.2 × 10^2^	2.8–3.6	3.0–7.8	9.5 × 10^−1^–2.8
	7.2–7.8	25	(5.9–9.1) × 10^−1^	6.5 × 10^−1^–1.6	(2.4–9.8) × 10^−1^

Yb	(1.4–1.8) × 10^2^	1.4 × 10^2^	30–32	24–29	12–24
	48–53	30	6.2–7.9	6.1–7.3	3.8–8.5

Lu	21–28	1.4 × 10^2^	4.4–6.1	2.8–7.2	2.7–6.3
	7.0–9.0	28	9.0 × 10^−1^–1.6	7.1 × 10^−1^–1.5	7.6 × 10^−1^–2.3

U	(1.4–1.7) × 10^3^	1.3 × 10^3^	(1.4–1.8) × 10^3^	(1.0–1.5) × 10^3^	(1.8–2.5) × 10^3^
	(4.6–5.0) × 10^2^	2.7 × 10^2^	(2.9–4.8) × 10^2^	(2.4–4.2) × 10^2^	(4.9–8.2) × 10^2^

*The concentrations of elements are expressed on the basis of both *ash weight* (the upper line) and dry weight (the lower line) of each brown algae species.

**Table 2:** Alginate content (%) in dry *S. h.* and *U. p.* samples.

Species	Alginate content (%)
*S. h.*(*n* = 2)	43.9 ± 4.1
*U. p.*(*n* = 2)	48.3 ± 2.6

**Table 3:** The average concentration factor of La, Eu, Yb, or U in *S. h.* and *U. p.*

	La	Eu	Yb	U
*S. h.*	2.4 × 10^3^	9.2 × 10^2^	2.8 × 10^3^	1.4 × 10^2^
*U. p.*	1.8 × 10^3^	7.9 × 10^2^	2.4 × 10^3^	2.5 × 10^2^

**Table 4:** Adsorption capacity (nmol g^−1^/dry wt) of La and U on each sample.

	*S. h.*	*U. p.*	Dry *U. p.*	Alginic acid
La	4.69	4.56	5.03	5.01
U	5.61	5.56	4.76	8.35
